# MCR-ALS-based muscle synergy extraction method combined with LSTM neural network for motion intention detection

**DOI:** 10.3389/fnbot.2023.1174710

**Published:** 2023-06-02

**Authors:** Dazheng Zhao, Yehao Ma, Jingyan Meng, Yang Hu, Mengqi Hong, Jiaji Zhang, Guokun Zuo, Xiao Lv, Yunfeng Liu, Changcheng Shi

**Affiliations:** ^1^School of Mechanical Engineering, Zhejiang University of Technology, Hangzhou, China; ^2^Ningbo Institute of Materials Technology and Engineering, Chinese Academy of Sciences, Ningbo, China; ^3^Ningbo Cixi Institute of Biomedical Engineering, Ningbo, China; ^4^School of Robotics, Ningbo University of Technology, Ningbo, China; ^5^Ningbo Ninth Hospital, Ningbo, China

**Keywords:** electromyographic signal, neural network, muscle synergy, joint angle detection, robustness

## Abstract

**Introduction:**

The time-varying and individual variability of surface electromyographic signals (sEMG) can lead to poorer motor intention detection results from different subjects and longer temporal intervals between training and testing datasets. The consistency of using muscle synergy between the same tasks may be beneficial to improve the detection accuracy over long time ranges. However, the conventional muscle synergy extraction methods, such as non-negative matrix factorization (NMF) and principal component analysis (PCA) have some limitations in the field of motor intention detection, especially in the continuous estimation of upper limb joint angles.

**Methods:**

In this study, we proposed a reliable multivariate curve-resolved-alternating least squares (MCR-ALS) muscle synergy extraction method combined with long-short term memory neural network (LSTM) to estimate continuous elbow joint motion by using the sEMG datasets from different subjects and different days. The pre-processed sEMG signals were then decomposed into muscle synergies by MCR-ALS, NMF and PCA methods, and the decomposed muscle activation matrices were used as sEMG features. The sEMG features and elbow joint angular signals were input to LSTM to establish a neural network model. Finally, the established neural network models were tested by using sEMG dataset from different subjects and different days, and the detection accuracy was measured by correlation coefficient.

**Results:**

The detection accuracy of elbow joint angle was more than 85% by using the proposed method. This result was significantly higher than the detection accuracies obtained by using NMF and PCA methods. The results showed that the proposed method can improve the accuracy of motor intention detection results from different subjects and different acquisition timepoints.

**Discussion:**

This study successfully improves the robustness of sEMG signals in neural network applications using an innovative muscle synergy extraction method. It contributes to the application of human physiological signals in human-machine interaction.

## 1. Introduction

Stroke is the main reason for death and disability among the elderly in China (Sacco et al., 2013). Most stroke patients suffer from motor dysfunctions, which seriously affects their ability to take care of themselves and imposes a heavy economic burden on society and families. Rehabilitation therapy can effectively help stroke patients rebuild limb movement function, but there is a serious shortage of rehabilitation therapists in China, and there are widespread problems such as high manual costs, low efficiency, and slow promotion. Compared with manual rehabilitation therapy methods, the convenient and efficient rehabilitation robot-assisted therapy is gradually showing the advantages of high repetition rate and tirelessness.

In the process of rehabilitation with robot-assisted training, human-robot interaction (HRI) is one of the key core technologies that determine the effectiveness of rehabilitation. The traditional program-based HRI approach has shackled the autonomous adaptive capability of robots and is difficult to apply to robotic systems that require direct integration with the human body. This has led to a new class of bioelectric signal-based HRI technologies. The core of the myoelectric signal-based HRI process lies in decoding human movement intention through myoelectric signals (Bi et al., 2019), and surface electromyographic signals (sEMG), as a non-invasive biofeedback pathway, have attracted a lot of attention from scientists in recent decades (Chowdhury et al., 2013; Tang et al., 2014; Ding et al., 2016).

Many studies that have used sEMG signals as a source of control signals for rehabilitation robots have focused on classification models, such as the simple sEMG-based two-finger opening and closing actions performed by Battye et al. (1955). And a five-finger pressing action activation model proposed by Peleg et al. (2002). However, this classification model is only capable of detecting pre-defined types of movements and rarely takes into account the non-ideal situations encountered when a person performs a task (Ma C. F. et al., 2021). As a result, the estimation of continuous movements is more practical.

Compared to building complex skeletal muscle models for motion detection, regression models using neural networks to establish the mapping relationship between sEMG signals and continuous joint motion are more concise and effective. For example, Sartroi et al. mapped elbow joint angles based on back propagation neural networks (Sartori et al., 2012). In 2017, Zhang et al. used artificial neural networks (ANN) for mapping and estimated the four joint angles of shoulder and elbow joints consecutively (Zhang et al., 2017). However, the neural network regression model suffered from the problem of over-reliance on the learned samples, and the detection values fluctuated when there were differences between the test and learned samples (Gers et al., 1999). The inherent instability and individual variability of the sEMG signal lead to high variability when it is used as a learning sample. The long-short term memory network (LSTM) has better performance for the case where the input data itself is temporal in nature and helps to eliminate the variation between samples. Due to the specificity and shareability of muscle synergy among the same actions (d'Avella and Bizzi, 2005; Ding et al., 2014), decoding the sEMG signal into muscle synergy features is beneficial to ensure the similarity between the testing signals and training signals of the neural network learning, thus enhancing the robust-ness of the detection results (Bizzi et al., 2008). The decomposition of muscle synergy determines the accuracy of the information contained in the decoded sEMG signal. However, neural networks learning using the synergy features derived from non-negative matrix factorization (NMF) as input can lead to low accuracy when training and testing samples obtained from different subjects and days (Gui et al., 2016), a new muscle synergy extraction method combined with LSTM to improve the robustness of motion intention detection was developed in this study.

Based on the above analysis, this study aims to investigate the accuracy and robustness of a reliable muscle synergy decomposition method in motion intention detection based on sEMG signals and LSTM neural networks. The multivariate curve resolution alternating least squares (MCR-ALS) method was used to extract muscle synergy as a feature mapping the angle of continuous elbow motion to construct a model for detection, and the muscle synergy features extracted by the traditional NMF method and PCA method were used as a comparison to validate the new method's accuracy and robustness in the detection of muscle synergy. The synergy features were used as a comparison to verify the robustness of the MCR-ALS method in different time periods and among different individuals. The robustness of motion intention detection of two methods was also compared to demonstrate the advantage of the proposed method.

## 2. Results

### 2.1. Results of acceleration calculation angle

**Figure 1:**
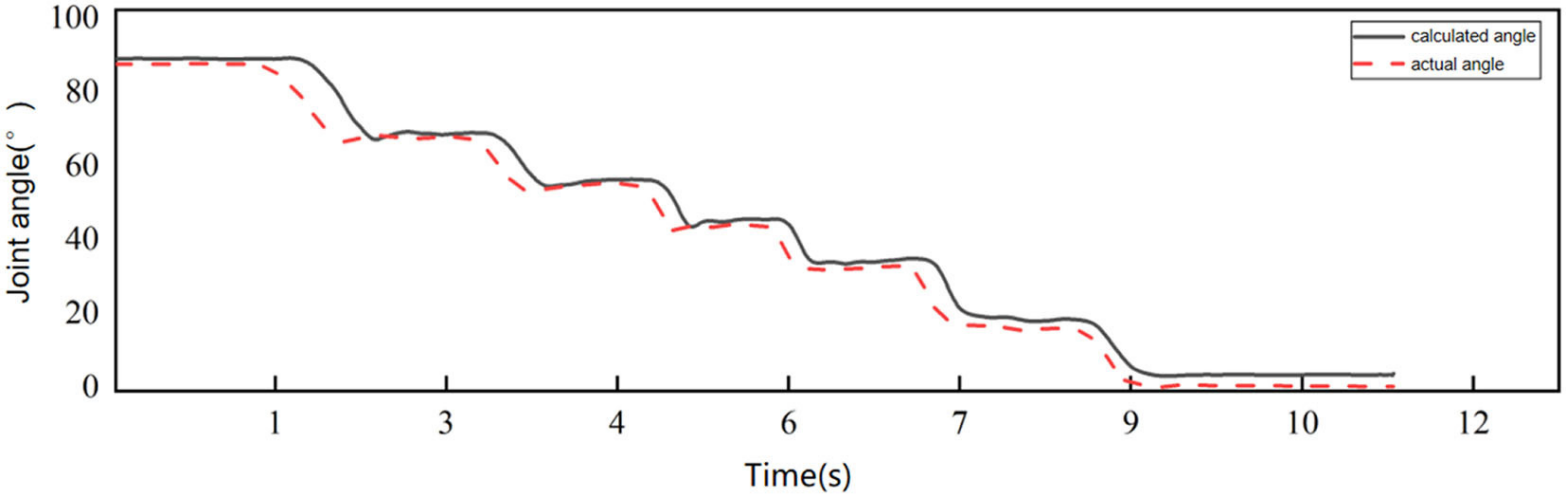
Comparison of calculated angle and actual angle.

In order to prove the accuracy of the calculation results, the calculation results are compared with the actual angle. The acceleration sensor and angle sensor were fixed at the same position on the upper arm of the subject, and the upper arm of the subject was required to remain motionless while the forearm moved from 0° to 90°. Figure 1 shows the comparison between the acceleration calculation results and the actual angle, and the mean absolute error (MAE) is used to measure the accuracy of the calculation.

The mean absolute error between the angle calculated by acceleration and the actual angle is 3.2415 ± 2.9440°, which meets the requirements of this study.

### 2.2. Results of motor function evaluation by muscle synergy

Considering that the sEMG signal amplitude of males is generally larger than that of females (Anders et al., 2004), the sEMG data of subjects with angular data were divided into two groups of male and female to train the models. The sEMG data from the first day of each group of all subjects were decomposed into MCR features, NMF features and PCA features to train Bi-LSTM neural network models, respectively, where 70% of the data were used as the training set and the remaining 30% were used as the test set. For the task-related component (Jiang et al., 2021), the fusion network that contained information from different people helped to eliminate interindividual variability.

The mean values of the correlation coefficients of muscle synergy obtained by the three algorithms were calculated separately for each subject over 5 days. The muscle synergy matrix obtained from each of the three algorithms for each participant per day was separately calculated. The correlation coefficients of all muscle synergy matrices obtained within a 5-day period for each participant were then computed and averaged. The Pearson Correlation Coefficient (CC) of muscle synergy obtained by the MCR-ALS algorithm were 0.9396 + 0.08, 0.8592 + 0.06, 0.8048 + 0.09, 0.9537 + 0.02, 0.9578 + 0.02, 0.9100 + 0.03, 0.9074 + 0.10, 0.8595 + 0.06, 0.9383 + 0.02, 0.9219 + 0.05, 0.9155 + 0.06, and 0.9178 + 0.01. The CC of muscle synergy obtained by the NMF algorithm were 0.4643 + 0.69, 0.0242 + 1.04, −0.8074 + 0.13, and −0.1694 + 0.84, 0.3372 + 0.58, 0.4779 + 0.71, 0.0713 + 1.0, 0.1003 + 0.81, 0.1461 + 0.92, 0.3771 + 0.46, 0.3540 + 0.87, and 0.2253 + 0.77. The CC of muscle synergy obtained by the PCA algorithm were 0.9143 + 0.07,0.8918 + 0.08, 0.8702 + 0.1, 0.9074 + 0.05, 0.9631 + 0.03, 0.7093 + 0.02, 0.9674 + 0.02,0.9616 + 0.03,0.9722 + 0.02,0.9577 + 0.02,0.8653 + 0.1,0.8729 + 0.09. The correlation coefficients of the three methods have significant differences (*F* = 17.027, *P* < 0.001).

**Figure 2:**
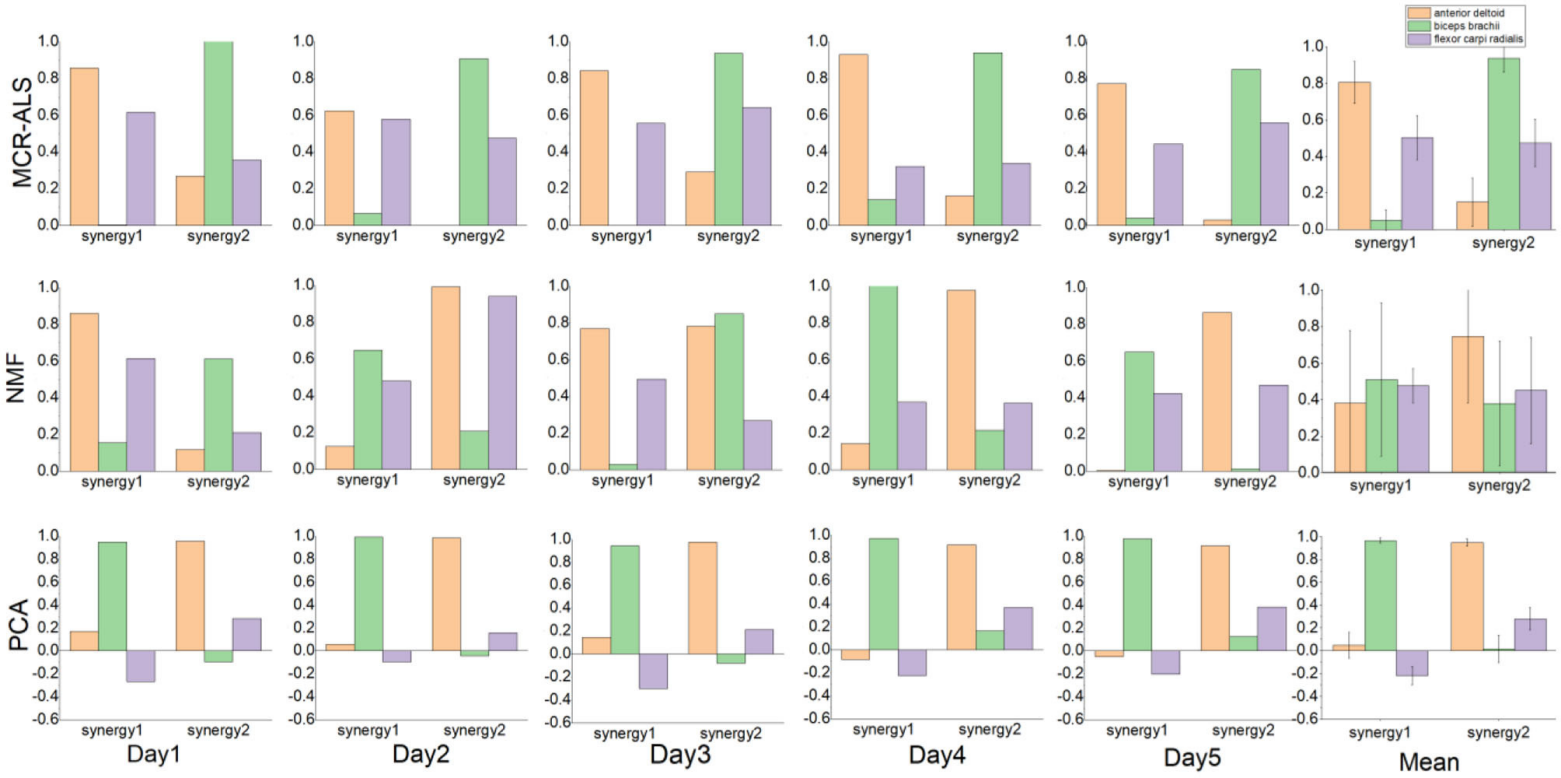
Comparison of the muscle synergy derived from the three methods.

A comparison of the muscle synergy matrices decomposed by the three decomposition methods for a particular subject over a 5-day period is shown in Figure 2. The superposition of the synergy matrix represents the synergistic fit pattern of muscle activation during the action, and the higher similarity of the synergy matrix represents the higher similarity of the extracted activation coefficient matrix, which is more stable as the input to the neural network. Figure 2 shows that the muscle synergies derived from the MCR-ALS method are stable throughout the duration of the experiment, while the muscle synergies decom-posed by the NMF are not in a fixed order and have poor similarity. For the PCA method, the extracted muscle synergies are relatively stable. However, due to the lack of non-negative constraints, the consistency of the synergies extracted by PCA and the other two methods is poor. The difference in synergy patterns may indicate that PCA may not fully reflect the corresponding synergy patterns of the action, which also affects the prediction results of neural networks.

**Figure 3:**
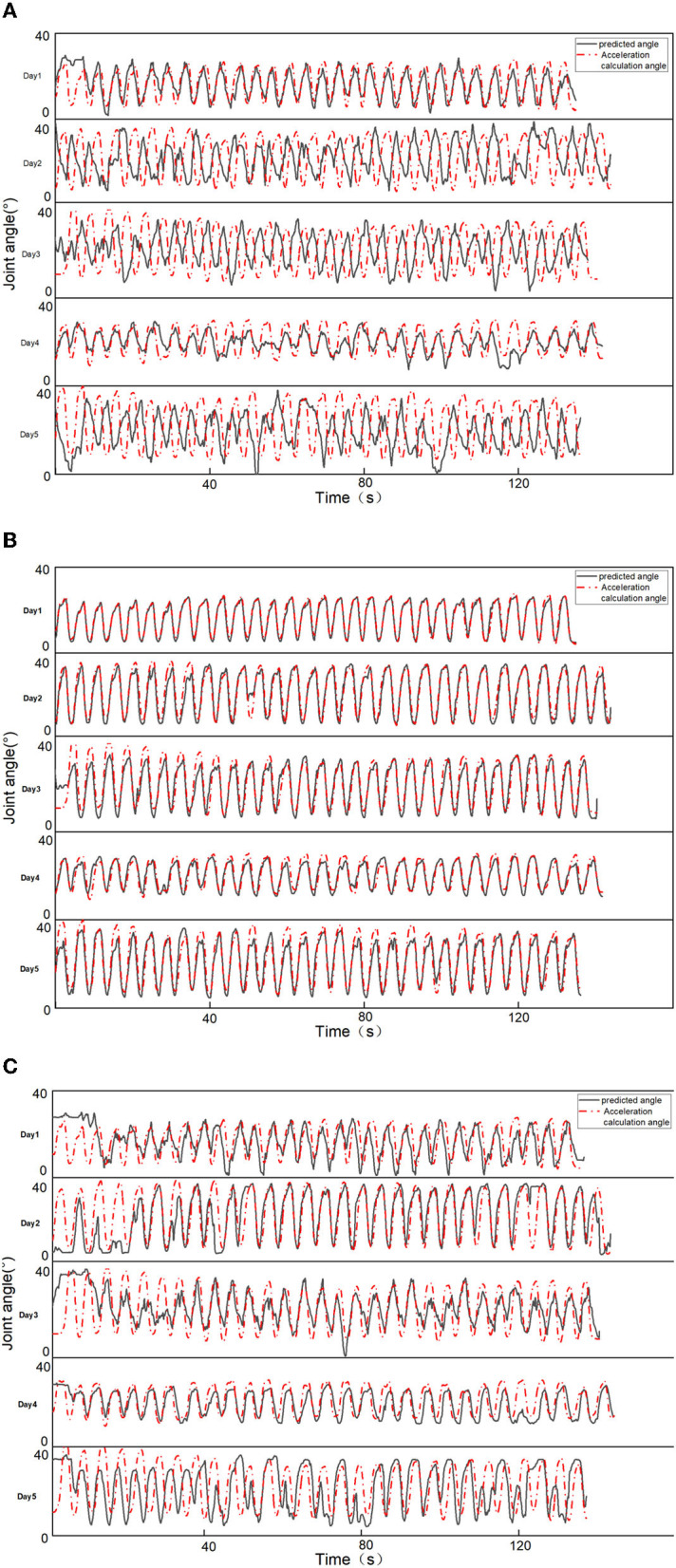
Comparison of prediction results of two features. **(A)** NMF feature prediction results. **(B)** MCR-ALS feature prediction results. **(C)** PCA feature prediction results.

To verify the robustness of the prediction results, compared the angular detection values of the three features with the actual values for a representative subject over 5 days. As shown in Figure 3, due to the reliable matrix decomposition performed by the MCR-ALS method, it can be found that the detection results of the model trained with the MCR features are more accurate and have robustness over a certain period of time, while the detection results of the NMF features are unstable and less accurate. The detection results of PCA features were also relatively stable, but the accuracy was poor, lower than that of MCR-ALS features. Figure 3 shows that the detection results of both methods maintain high agreement with the actual angle on the first day. From the second day, the detection results of NMF features start to become less consistent with the actual ones, while the consistency of the results of MCR-ALS features stays above 85% for the next 4 days. The accuracy of PCA features was consistently and significantly lower than that of MCR-ALS features over a period of 5 days. In the case of NMF features, the detection results were inconsistent, with better and worse performance observed on different days. However, on days where the results were ideal, such as the first and fourth days, the predictive accuracy of NMF features was higher than that of PCA features, although both were inferior to MCR-ALS features.

To verify the accuracy of the results, the correlation coefficients between the test results and the actual measurements were calculated for both groups of male and female subjects as shown in Tables 1, 2.

**Table 1:** Comparison of the accuracy of three methods for male subjects (*N* = 7).

	**MCR-ALS**	**NMF**	**PCA**
	**Mean** ±**standard deviation**	**Mean** ±**standard deviation**	**Mean** ±**standard deviation**
Subject1	0.8794 ± 0.06	0.3382 ± 0.14	0.4399 ± 0.08
	0.9148 ± 0.04	0.4763 ± 0.29	0.5186 ± 0.12
	0.8666 ± 0.01	0.3596 ± 0.15	0.4374 ± 0.07
Subject2	0.8088 ± 0.08	0.5270 ± 0.04	0.4087 ± 0.09
	0.8223 ± 0.01	0.5345 ± 0.20	0.5758 ± 0.11
	0.8456 ± 0.03	0.4589 ± 0.14	0.4148 ± 0.06
Subject3	0.8393 ± 0.08	0.6385 ± 0.08	0.4980 ± 0.08
	0.8517 ± 0.08	0.3977 ± 0.28	0.4772 ± 0.09
	0.8240 ± 0.05	0.3734 ± 0.24	0.4618 ± 0.07
Subject4	0.8179 ± 0.03	0.5728 ± 0.09	0.5251 ± 0.15
	0.8398 ± 0.02	0.4755 ± 0.28	0.4233 ± 0.08
	0.8081 ± 0.04	0.3073 ± 0.26	0.4124 ± 0.14
Subject5	0.8758 ± 0.05	0.5249 ± 0.16	0.4349 ± 0.04
	0.8484 ± 0.03	0.4309 ± 0.20	0.4105 ± 0.04
	0.8981 ± 0.02	0.4071 ± 0.25	0.4665 ± 0.03
Subject6	0.8900 ± 0.04	0.2689 ± 0.11	0.6321 ± 0.01
	0.8593 ± 0.05	0.4871 ± 0.23	0.4009 ± 0.10
	0.8563 ± 0.02	0.3657 ± 0.24	0.2677 ± 0.05
Subject7	0.8608 ± 0.05	0.1830 ± 0.11	0.5577 ± 0.11
	0.8599 ± 0.04	0.3655 ± 0.19	0.3839 ± 0.09
	0.8506 ± 0.04	0.3310 ± 0.18	0.2865 ± 0.07

**Table 2:** Comparison of the accuracy of three methods for female subjects (*N* = 5).

	**MCR-ALS**	**NMF**	**PCA**
	**Mean** ±**standard deviation**	**Mean** ±**standard deviation**	**Mean** ±**standard deviation**
Subject1	0.8920 ± 0.04	0.2992 ± 0.13	0.4139 ± 0.07
	0.8425 ± 0.05	0.3182 ± 0.16	0.4866 ± 0.10
	0.8410 ± 0.03	0.3176 ± 0.15	0.3214 ± 0.06
Subject2	0.8888 ± 0.06	0.2713 ± 0.13	0.3429 ± 0.05
	0.8449 ± 0.05	0.5583 ± 0.19	0.6425 ± 0.09
	0.8572 ± 0.05	0.4614 ± 0.23	0.6309 ± 0.10
Subject3	0.8670 ± 0.10	0.1395 ± 0.09	0.3607 ± 0.14
	0.8338 ± 0.08	0.3938 ± 0.16	0.4200 ± 0.04
	0.8523 ± 0.01	0.3362 ± 0.14	0.3224 ± 0.04
Subject4	0.8858 ± 0.04	0.2575 ± 0.22	0.4426 ± 0.10
	0.8716 ± 0.02	0.3362 ± 0.14	0.3962 ± 0.05
	0.8491 ± 0.01	0.3975 ± 0.17	0.3738 ± 0.04
Subject5	0.8132 ± 0.10	0.2980 ± 0.19	0.4108 ± 0.08
	0.9041 ± 0.02	0.3997 ± 0.16	0.4372 ± 0.09
	0.8830 ± 0.02	0.3780 ± 0.16	0.1723 ± 0.07

## 3. Discussion

The MCR-ALS decomposition method and the traditional NMF synergy decomposition method were compared and analyzed. Since the initial matrix in the NMF decomposition was built randomly, the muscle synergy order and amplitude derived after iteration were not fixed, which caused the differences in the input variables of the neural network and affected the accuracy and stability of the output results. In contrast, the muscle synergy matrix derived from the MCR-ALS decomposition method is relatively stable each time, which provides excellent sEMG signal feature information for the Bi-LSTM neural network motor intention detection. The experimental results showed that this decomposition method has high accuracy for both male and female detection. In comparison, PCA features are also obtained by dimensionality reduction with a determinate matrix of base vectors, resulting in a certain degree of stability in the detection results. However, the PCA method can only provide linear transformations of the data set, and is more sensitive to noise and outliers. Therefore, when processing physiological data sets, the PCA method often fails to accurately identify the true muscle coordination patterns, which leads to lower detection results for PCA features than for MCR-ALS and NMF features.

The Bi-LSTM neural network can extract useful information in both temporal directions, and its unique memory mechanism can retain the association between various time points in a longer time sequence. Since muscle synergy of the same action is consistent, this information retention was helpful for the neural network that uses synergy as a feature to detect actions over a period of time, enhancing the robustness of sEMG signals over time. From the experimental results, it can be seen that the accuracy of the detection results was around 80% on all 5 days.

During the experiment, before gender division, we extracted muscle synergy features by combining the 3-channel EMG signals of all 12 participants (the sample size was limited) to train a model. However, the predicted results were lower than expected for both male and female participants, despite being able to predict the specific angle change waveform. The maximum and minimum values of angle change predicted for each movement were also less accurate compared to actual angle changes. After gender division, the angle detection results for both male and female groups improved by about 10%. This phenomenon may be different physiological structures of men and women, men have more muscle cells and the signal amplitude of muscle activation was larger when completing the same action. During the training process of the network, this would lead to excessive differences between the training set data and the training effect. Dividing the subjects into two groups of men and women can exclude the gender difference in the sEMG signals and reduce the error of experimental results.

Two separate models were trained for the male and female groups, each using data from all subjects on the first day only. The models incorporate information from different subjects, and the resulting models are transferable to all subjects in the group and robust to the effects of electrode displacement and sEMG signal changes over time over 5 days. It helps to exclude confounding factors of the sEMG signal under non-ideal conditions.

## 4. Conclusion

In this paper, the muscle co-activation coefficient matrix obtained by the muscle co-extraction method based on MCR-ALS is used as the sEMG signal features, and the movement estimation is performed in a Bi-LSTM neural network with an upper limb motion angle mapping training model. This study provides conditions for the utilization of human electrophysiological signals during the HRI of rehabilitation robots. It contributes to the promotion of HRI technology in a series of application scenarios such as assisted rehabilitation robots and rehabilitation assistive devices, so as to lay the foundation for the subsequent application of new HRI technology based on bioelectrical signals from laboratory scenarios to real-life applications. However, there are still some limitations to this study. The experimental movements were limited to simple elbow flexion movements with fixed trajectories, which limited the flexibility of the model. The future work will focus on extending the motion intent detection to more complex multi-joint movements for the applications in clinical or practical conditions.

## 5. Methods

### 5.1. Muscle synergy theory

Muscle synergy can explain how the central nervous system (CNS) accomplishes complex movements (Cheung et al., 2012). The CNS coordinated the activation of certain muscle synergies to perform motor tasks. For example, a single muscle can be part of multiple muscle synergies, and a single muscle synergy can be composed of multiple muscles. Muscle synergy effectively represents the collaborative activation of muscles during a task.

In muscle synergy theory, the sEMG signal is decomposed into an activation coefficient matrix containing information about the muscle activation time and a synergy matrix reflecting the relative activation intensity of multiple muscles. Thus, the activity state of a muscle can be represented as a linear combination of the synergy matrix and the muscle activation coefficient matrix (Shourijeh et al., 2016).


(1) $$\begin{array}{l} \text{D}=\text{CS}+\text{E} \end{array}$$

where *D* is the *m* × *n* matrix, *m* denotes the number of myoelectric signal channels, and *n* denotes the length of the time series corresponding to each myoelectric signal channel; *C* is the *m* × *r* muscle synergy matrix, reflecting the relative activation strength of multiple muscles; *r* denotes the number of muscle synergies; *S* is the *r* × *n* activation coefficient matrix; and *E* is the error matrix of size *m* × *n*.

### 5.2. LSTM neural network

Recurrent neural networks (RNNs) have been widely used in the study of sequential data such as text, audio, and video (Werbos, 1988; Horii et al., 2020). However, traditional RNNs can only use in-formation from a certain period of time ago. And when the input gap is large, recurrent neural networks using only activation functions like sigmoid or tanh are prone to gradient explosion or gradient disappearance.

**Figure 4:**
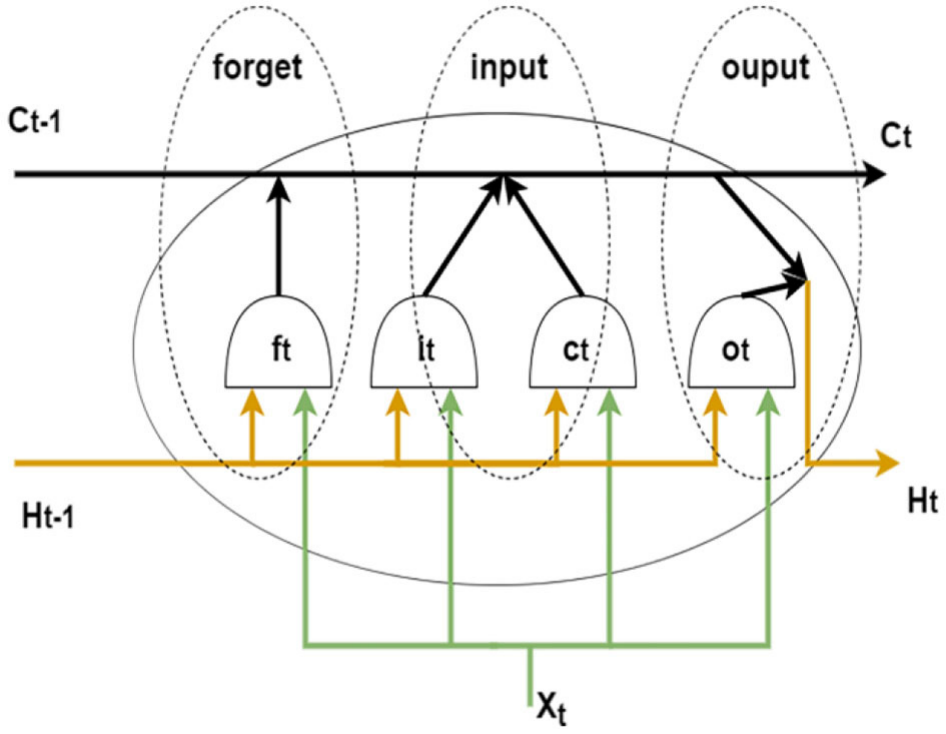
LSTM cell structure.

Hochreiter and Schmidhuber proposed long and short-term memory networks in 1997 (Hochreiter and Schmidhuber, 1997). By introducing gate functions in the cell structure, long and short-term memory networks can deal well with long-term dependencies. Graves and Schmidhuber combined the bidirectional RNN introduced by Schuster and Paliwal (1997) with LSTM cells to propose a bidirectional LSTM (Bi-LSTM) (Graves and Schmidhuber, 2005). This structure can be trained in two temporal directions simultaneously with separate hidden layers (i.e., forward and backward layers). Figure 4 shows the cell structure of LSTM, each forward layer is connected by stacked LSTM cells. The forward layer outputs are ordered from *t* = 1 to *t* = *T*. And the backward layer outputs are ordered from *t* = *T* to *t* = 1. The information in both directions effectively extended the content referenced by the LSTM network.

### 5.3. Experimental data acquisition

**Figure 5:**
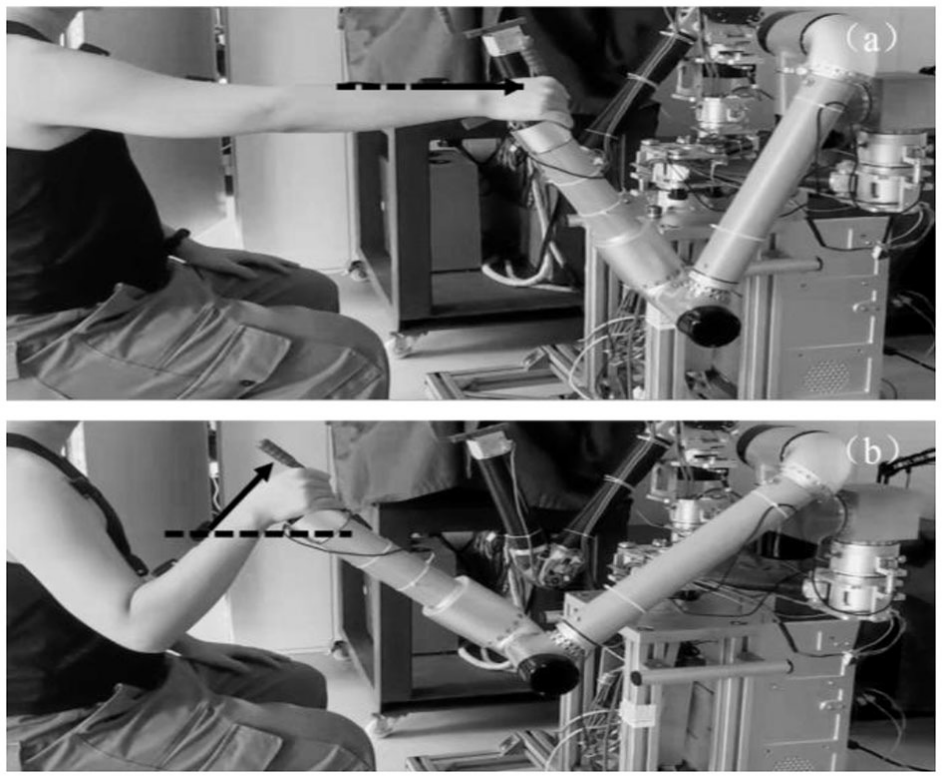
Experimental action design. **(a)** Elbow extension. **(b)** Elbow flexion.

Twelve healthy subjects (seven males and five females, mean age was 24 years) completed elbow flexion and extension trials along a reference trajectory guided by a training game in the same time period for five consecutive days. This work involved human subjects in its research. Approval of all ethical and experimental procedures and protocols was granted by the Ethic Committee of Ningbo Institute of Materials Technology and Engineering, Chinese Academy of Science and performed in line with the Declaration of Helsinki. All subjects signed and gave the written informed consent forms to participate the experiments. As shown in Figure 5, this experiment was performed on a bilateral mirror image rehabilitation platform developed in our laboratory, limiting the arm trajectory to ensure similarity of repetitive movements. on the first day, subjects completed two sets of 30 trials each, with a 2-min rest between sets. One set of data was used as the training set to train the network, and the other set was used as the test set to test the network performance. On the second 4 days, subjects completed one set of 30 trials each day, and the data was used as a validation set to verify the accuracy of the network detection results.

**Figure 6:**
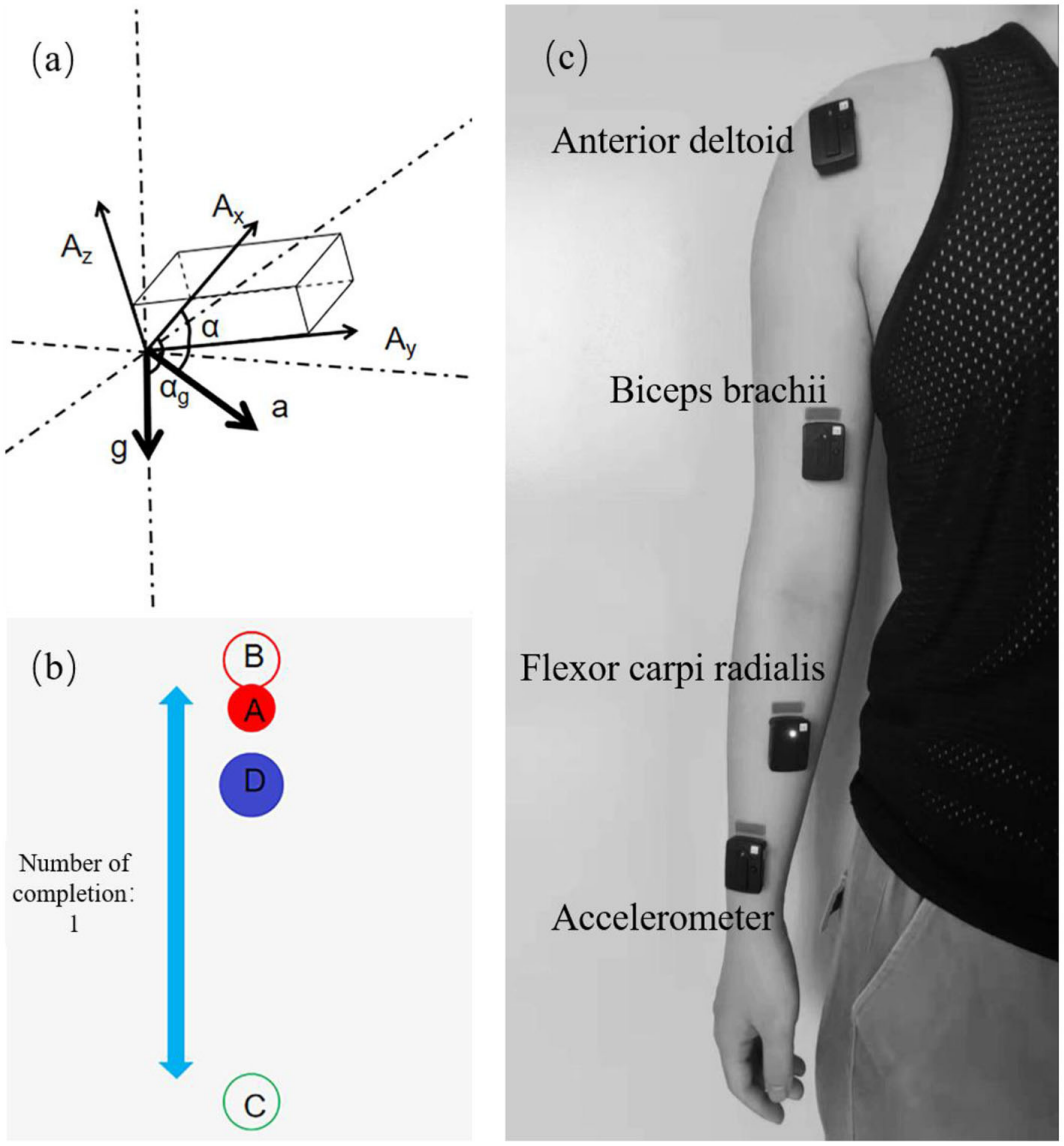
Experimental design and data collection. **(a)** Principle of angular velocity calculation. **(b)** Training game interface. **(c)** Sensor position distribution.

Figure 6b showed the interface of the training game based on the Unity3D platform. The red ball A represent the original reference position at the end of the robot arm; the red circle B and green circle C represent the path points of the reference trajectory; and the blue ball D represent the task target to be tracked. The blue ball moves back and forth between the red and green circles at a constant speed.

The sEMG acquisition device used in this experiment was the Trigno Wireless EMG System from DELSYS, U.S.A. The device consists of 16 sEMG signal sensors and a wireless receiving base station, which can simultaneously acquire 16 channels of sEMG signals with a sampling rate of up to 2,000 Hz. Three muscles of the subject were selected, which were the anterior deltoid (DA), biceps brachii (BI), and flexor carpi radialis (FCR). After disinfecting with alcohol pads and exfoliating with facial scrub, the three sEMG sensors were attached along the muscle texture in the order shown in Figure 6c.

To ensure the synchronization of the sEMG signal and the angle signal, the angle in-formation is calculated using the acceleration module in the Trigno sEMG acquisition system after measuring the acceleration.

### 5.4. Muscle synergy extraction

Principal component analysis (PCA) is a common data dimensionality reduction technique used to extract the most representative principal components from high-dimensional data. As shown in Eq. 2, first, the PCA method computes the covariance matrix *V* of the standardized raw matrix D of the electromyographic signals, and performs an eigendecomposition of the covariance matrix to obtain the eigenvector matrix *V* and the eigenvector value vector λ. The eigenvectors are then sorted in descending order of their corresponding eigenvalues, and the top k eigenvectors are selected as the new base vectors, which yield the activation patterns of each muscle in each synergy, i.e., the muscle synergy matrix *C*. The temporal information of each synergy can be obtained by computing the reduced data matrix *S*, as shown in Eq. 3.


(2) $$\begin{array}{l} V=\frac{1}{\text{m}-1}{\left(D-\bar{D}\right)}^{T}\left(D-\bar{D}\right) \end{array}$$

(3) $$\begin{array}{l} S=DC \end{array}$$

Non-negative matrix decomposition is the most widely used method for muscle coextraction extraction from sEMG signals (Gunay et al., 2017; Hu et al., 2021; Ma Y. H. et al., 2021). The muscle synergy matrix is generated by iteratively optimizing a randomly created initial matrix by minimizing the Frobenius parametrization of the residual matrix in Eq. 4. *Q* in Eq. 5 is the underfitting transformation rate between two iterations and determines the stopping criterion of the iteration. The iterations are stopped when *Q* equals 0.01% or when the number of iterations reaches 1,000.


(4) $$\begin{array}{l} f\left(S,C\right)=\frac{1}{2}{∥D-CS∥}_{F}^{2} \end{array}$$

(5) $$\begin{array}{l} Q=100\cdot \left(\frac{f_{l+1}\left(S,C\right)-f_{l}\left(S,C\right)}{f_{l}\left(S,C\right)}\right) \end{array}$$

MCR-ALS is a commonly used matrix decomposition algorithm for resolving the component information in hybrid systems (Yu et al., 2019). In this study, the initial matrix of MCR-ALS was obtained by Self Modeling Mixture Analysis (SMMA). The SMMA method determined the pure variable matrix by calculating the pure variable values of the signal samples and finding the maximum pure variable value. The initial matrices *C* and *S* for the decomposition of the sEMG signal D are calculated using the pure variable matrices (Windig and Stephenson, 1992).

Self-modeling mixture analysis (SMMA), also known as simple-to-use interactive self-modeling mixture analysis (SIMPLISMA), is a linear unmixing method used to analyze mixture systems. The method is based on the concept of pure variables, which are variables that contribute to only one component of the system. In muscle synergy analysis, pure variables refer to the sEMG data points that are associated with the recruitment of only one synergy, meaning they are sampled in isolation from other synergies. Thus, SMMA is a useful technique for decomposing complex mixture systems into their pure variable components.

The pure variables are based on variables having the ratio of the maximum standard deviation to the mean. As shown in Eq. 6.


(6) $$\begin{array}{l} p_{ij}=\omega_{ij}\frac{{\hat{\sigma }}_{i}}{{\hat{\mu }}_{i}+\alpha } \end{array}$$

where *p*_*ij*_ is the value of the ith pure variable, from which the jth pure variable will be selected. and ${\hat{\mu }}_{i}$ ${\hat{\sigma }}_{i}$ are the mean and standard deviation of variable *i*. α is a constant in the range of 1%−5% and is added to the denominator to avoid the problem of variable level noise averaging. The weight factor ω_*ij*_, a determinant-based function, is used to correct for the previously selected pure variables.


(7) $$\begin{array}{l} \mu_{i}=\left(1/m\right)\overset{m}{\underset{j=1}{\sum }}d_{ij} \end{array}$$

(8) $$\begin{array}{l} \sigma_{i}={\left(\left(1/m\right)\overset{m}{\underset{j=1}{\sum }}{\left(d_{ij}-\mu_{i}\right)}^{2}\right)}^{1/2} \end{array}$$

where *d*_ij_ is the element matrix of *D* with size of *m* × *n*, which composes m sEMG signals, and each sEMG signal has n samples.

For the same data, the NMF initialization was random, so its solution had non-uniqueness. Moreover, it was easy to fall into local optimum in the decomposition process, which aggravated the difference between the test and learning samples and significantly affected the neural network's detection for the results. Compared with NMF, MCR-ALS has better repeatability and consistency because the initial matrix is calculated based on the SMMA method and the initial matrix is consistent for each decomposition of the same data (Matsunaga et al., 2021).

According to Eq. 6, Once the pure variables have been determined, their intensities are extracted to form synergy matrix C, then the sEMG data set can be unmixed through matrix transformation.

In addition, alternating least squares (ALS) was used to optimize the initial resolution according to Eqs 9, 10. During the iterative process, non-negative constraints are imposed. The stopping criterion of MCR-ALS was the same as that of NMF.


(9) $$\begin{array}{l} C=\left(DS^{T}\right){\left(SS^{T}\right)}^{-1} \end{array}$$

(10) $$\begin{array}{l} S={\left(C^{T}C\right)}^{-1}C^{T}D \end{array}$$

### 5.5. Data processing and neural network mapping

The EMG signal *D* was recorded from the subject. After the preprocessing and feature extraction of the sEMG signal, the activation coefficient matrix S_NMF_ and the muscle synergy matrix *C*_NMF_ were decomposed by Eqs 4, 5, and the activation coefficient matrix *S*_MCR_ and the muscle synergy matrix C_MCR_ were decomposed by Eqs 9, 10. Where *S*_NMF_ and *S*_MCR_ are *r* × *n* activation coefficient matrices, and the number of synergies r is explained by variance account for (VAF). The VAF reflected the degree of similarity between the decomposed reconstructed data and the original data. The minimum number of synergies that can reconstruct the EMG data was chosen under the condition that the VAF value be >80% (Li et al., 2017). VAF was calculated according to the following equation:


(11) $$\begin{array}{l} VAF=1-\left({\left||D-M|\right|}^{2}/{\left||D-mean\left(D\right)|\right|}^{2}\right) \end{array}$$

The reconstruction matrix of the synergy extraction algorithm is represented by M = CS, where M has the same size as *D*. The “mean” operator produces a matrix with columns that are the mean of the corresponding columns in *D*. In this experiment, the number of synergies was chosen as 2.

The length of time series *n* indicated the length of sEMG signals from three channels during the duration of a set of experiments.


(12) $$\begin{array}{l} \text{D=}\left[{\text{D}}_{\text{1}}\;{\text{D}}_{\text{2}}\;{\text{D}}_{\text{3}}\right] \end{array}$$

As shown in Figure 6a, the output of the sensor XYZ triaxis is determined by both the external force and gravity (Wen-sheng et al., 2009). It is assumed that the sensor outputs in the three axes are A_x_, A_y_, and A_z_, respectively, and the acceleration of gravity is g and the acceleration of the combined external force is a. Then the sensor outputs in the three axes directions should be


(13) $$\begin{array}{l} \text{a}\cos\;\alpha \;+\text{g}\cos{\;\alpha \;}_{\text{g}}={\text{A}}_{\text{x}} \end{array}$$

(14) $$\begin{array}{l} \text{a}\cos\;\beta \;+\text{g}\cos{\;\beta \;}_{\text{g}}={\text{A}}_{\text{y}} \end{array}$$

(15) $$\begin{array}{l} \text{a}\cos\;\gamma \;+\text{g}\cos{\;\gamma \;}_{\text{g}}={\text{A}}_{\text{z}} \end{array}$$

where α, β, γ denote the angle between the combined external acceleration of the sensor and the three axes of the sensor, and α_g_, β_g_, γ_g_ denote the angle between the acceleration of gravity and the three axes of the sensor.

In the elbow flexion action shown in Figure 5, the acceleration of the sensor in the x-axis and y-axis direction was 0, and the output value was only affected by the acceleration of gravity. Therefore, the joint motion angle θ can be regarded as the angle between the sensor x-axis and the horizontal direction, which is the residual angle of α_g_.


(16) $$\begin{array}{l} \;\theta \;=90^{•}-\text{arccos}\frac{{\text{A}}_{\text{x}}}{\text{g}} \end{array}$$

To exclude errors, the acceleration sensor is calibrated by gravity and the acquisition results are low-pass filtered.

In the process of model training, the input of neural network was the 3-dimensional muscle cooperative activation coefficient matrix feature of EMG signal, and the output was the 1-dimensional angle information. The backpropagation strategy is used to train the network, and there were five Bi-LSTM layers, each with 48 hidden units. To prevent overfitting, a dropout layer is inserted after each Bi-LSTM layer, and the drop ratio is set to 0.3. After train the model with the experimental data on the first day, the model is used to make predictions on the last 4 days as a way to verify the robustness of the model in the time horizon.

### 5.6. Algorithm evaluation

The Pearson Correlation Coefficient (CC) was used to measure the similarity between the detection results and the actual measurement results to assess the accuracy of the model.


(17) $$\begin{array}{l} CC=\frac{\sum_{i=1}^{n}\left(\theta_{i}-\bar{\theta }\right)\left(\theta_{ri}-\bar{\theta_{r}}\right)}{\sqrt{\sum_{i=1}^{n}{\left(\theta_{i}-\bar{\theta }\right)}^{2}}\sqrt{\sum_{i=1}^{n}{\left(\theta_{ri}-\bar{\theta_{r}}\right)}^{2}}} \end{array}$$

where θ is the detection angle, θ_r_ is the actual measurement angle, and *n* is the length of the time series.

This study measured the stability of the decomposed muscle synergy by comparing the CC of muscle synergy over a certain time range. A paired *t*-test was used to measure the muscle synergy consistency between the two algorithms.

## Data availability statement

The raw data supporting the conclusions of this article will be made available by the authors, without undue reservation.

## Ethics statement

The studies involving human participants were reviewed and approved by Ethic Committee of Ningbo Institute of Materials Technology and Engineering, Chinese Academy of Science. The patients/participants provided their written informed consent to participate in this study.

## Author contributions

Conceptualization and writing—review and editing: XL, YL, and CS. Methodology: YM, DZ, and CS. Resource: XL, GZ, and CS. Software, formal analysis, investigation, and writing—original draft preparation: YM and DZ. Validation and data curation: JM and YH. Supervision: YL and CS. Visualization: MH, JZ, and GZ. Funding acquisition: GZ, XL, and CS. All authors have read and agreed to the published version of the manuscript.
